# “Olfactory Three-Needle” Enhances Spatial Learning and Memory Ability in SAMP8 Mice

**DOI:** 10.1155/2020/2893289

**Published:** 2020-01-02

**Authors:** Yuan Wang, Qiang Wang, Bo Ren, Ting Guo, Jing Qiang, Hui Cao, Yujie Gao, Zhibin Liu, Xinyan Gao, Bing Zhu

**Affiliations:** ^1^Institute of Acupuncture and Moxibustion, China Academy of Chinese Medical Sciences, Beijing 100700, China; ^2^College of Acu-moxibustion and Massage, Shaanxi University of Chinese Medicine, Xianyang 712046, China; ^3^Shaanxi Key Laboratory of Acupuncture and Medicine, Xianyang 712046, China

## Abstract

As one of the most important therapies in complementary and alternative medicine, acupuncture has been used in the treatment of Alzheimer's disease (AD). Acupuncture of “olfactory three-needle” manipulation can improve the cognitive ability of AD patients. However, the mechanism of “olfactory three-needle” in AD remains largely unknown. Here, we identified that the “olfactory three-needle” therapy and eugenol olfactory stimulation both reduced the deposition of *β*-amyloid (A*β*) protein and increased the expression of synaptophysin (SYP), but only the “olfactory three-needle” enhanced the spatial learning and memory ability of SAMP8. Remarkably, the “olfactory three-needle” inhibited the phosphorylation of p38MAPK and the excessive activation of microglia (MG) in the hippocampus. Our study demonstrates that the “olfactory three-needle” enhances spatial learning and memory ability by inhibiting the phosphorylation of p38MAPK and the excessive activation of MG to reduce the neuroinflammatory response and neurotoxicity of A*β* and promote synaptic regeneration, but it was not completely consistent with the stimulation of the olfactory system.

## 1. Introduction

Alzheimer's disease (AD) is an insidious, progressive, and neurodegenerative disease characterized by progressive memory loss, cognitive impairment, behavioral abnormalities, and decreased living ability [1]. With the aggravation of social aging, the incidence of AD has gradually increased, and extensive literature demonstrates that aging is accompanied by olfactory loss and hyposmia/anosmia, which is also a feature of several neurodegenerative disorders [2].

However, olfaction is frequently referred to as the “neglected” sense because it is rarely investigated in medical practice, where patients are often unaware of their olfactory problems.

Currently, no specific treatment exists to halt or reverse the AD, and most approaches are aimed at delaying the loss of function and preserving cognition and memory [3]. Curative treatment of AD can improve symptoms, but the pharmacological interventions have been associated with raised costs and increased risk of adverse events [4]. In recent years, acupuncture of the scalp, “olfactory three-needle” manipulation, and body has been widely used to improve mini mental state examination scores, activities of daily living, and the hierarchic dementia scale of AD patients, with fewer associated adverse events occurring [5]. Furthermore, the eugenol, extracted from several spices and medicinal herbs, has been shown to have a neurogenerative activity and have the broad effects on neuroprotection in several neurodegenerative diseases [6].

Our previous clinical research showed that the acupuncture treatment of “olfactory three-needle” had obvious therapeutic effects on olfactory dysfunction and cognitive dysfunction in AD patients [7]. Then, we investigated the effects of “olfactory three-needle” and eugenol on learning-memory ability and the antioxidation system of the hippocampus in AD rats, and we found that both “olfactory three-needle” and eugenol can stimulate the olfactory system to increase learning-memory ability, and the antioxidation system of the hippocampus in AD rats [8]. Nonetheless, the mechanism remains unknown. According to the local acupoint-taking principle, the “olfactory three-needle” manipulation acupunctures the acupoints of Yintang, bilateral, and Yingxiang, which are the important olfactory-related acupuncture point. Therefore, we hypothesize that the “olfactory three-needle” may be stimulating the olfactory system to improve the cognitive and memory abilities of AD patients.

Considering that neurotoxicity of A*β* plays the central role in neuroinflammatory response and initiation of the neurodegenerative process of AD [9]. It is vital to uncover how “olfactory three-needle” can impact the regulation of A*β* and neuroinflammatory in the AD model. In the current study, we compared the olfactory stimulation effects of “olfactory three-needle” and eugenol on the spatial learning and memory; the protein expression of *β*-amyloid (A*β*), phosphorylated p38 MAPK (P-p38 MAPK), phosphorylated tau protein (P-tau), and synaptophysin (SYP); and the activation status of microglia (MG) in the hippocampi of SAMP8 mice, to explore the mechanism of “olfactory three-needle” in improving AD cognitive and memory abilities. Our data represent a significant contribution to the identification of the effectiveness of “olfactory three-needle” in improving spatial learning and memory ability in a mouse model of AD.

## 2. Materials and Methods

### 2.1. Experimental Animals

Seven-month-old male (33 ± 5 g) senescence-accelerated prone mouse 8 (SAMP8 mice) and senescence-accelerated mouse resistance mice 1 (SAMR1) were purchased from the Experimental Animal Center of Military Medical Sciences of the PLA (Animal Lot: SCXK (Jing) 2014-0011). The mice were housed in the Animal Center of Acupuncture and Moxibustion Research Institute of Chinese Academy of Traditional Chinese Medicine at a controlled temperature (22 ± 2°C), 55% humidity, and under a 12 h dark/light cycle, with sterile drinking water and a standard pellet diet available *ad libitum*. All mice were acclimatized to the environment for 7 days prior to experimentation.

All animal experiments followed the guidelines established by the ethics committee for the Care and Use of Laboratory Animals in the College of Acu-moxibustion and Massage of Shaanxi University of Chinese Medicine and were approved with the permit numbers (AM2018-7041).

### 2.2. Open-Field Trial

We proceeded to an open-field trial before grouping in order to exclude unqualified mice in autonomous activities. Open-field trial video analysis system (Xinxin, Shanghai) mainly includes acquisition box, video acquisition device, and software system. The open-field collection box was four connected black uncovered wooden boxes of 50 × 50 × 50 cm in size, with the mice placed in and faced the wall of the box, testing for 5 min to observe the time and distance of the mice moving in the central area during the effective time, and thereby evaluated autonomous activity status of the mice. In addition, feces and other excreta in the box have to be cleaned after each test [10].

### 2.3. Animal Grouping and Intervention

After the open-field test, unqualified mice in autonomous activities were excluded. Thirty SAMP8 mice were divided into three groups (10 per group): the SAMP8 control (P8N) group, the SAMP8 acupuncture (P8A) group, and the SAMP8 olfactory stimulation (P8O) group. Ten SAMR1 mice were invoked as the normal control (R1N) group.

“Olfactory three-needle” intervention: the mice of the P8A group were anesthetized with isoflurane. According to the acupoint selection method in the attached drawings of experimental animal acupoints commonly used in the 9th edition of Experimental Acupuncture and Moxibustion, the disposable sterile acupuncture needles (0.3 mm × 13 mm) (Huatuo Medical Instrument Company, Suzhou) were used to puncture the Yintang (GV29) with transverse puncturing to the nasal root direction at a depth of 10 mm, and the bilateral Yingxiang (LI20) with shallowly puncturing towards the interior and superior at a depth of 2 mm. The bilateral Yingxiang (LI20) was stimulated with HANS-200 electric stimulator (Han Shi, Nanjing) for 10 minutes (current intensity: 1.5 mA, wave: 15 Hz).

Olfactory stimulation intervention: putting 1 ml eugenol solvent (Aladdin, USA) in the sealed laboratory container (20 cm × 20 cm × 60 cm, plastic box, self-made), smell of eugenol solvent diffused for 10 minutes, then the mice of the P8O group were kept in a box for olfactory stimulation, and taken out after 30 minutes. Intervention of “olfactory three-needle” and olfactory stimulation was administered 6 days a week for 4 consecutive weeks, with no treatment of the R1N or P8N groups. The above intervention lasted throughout the Morris water maze test period.

### 2.4. Morris Water Maze Test

Morris water maze tests were employed to evaluate the spatial learning and memory ability of the mice, and the protocols of the Morris water maze tests have been described in previous studies [8, 11]. Mice were trained to locate a hidden platform (7 cm in diameter, 1 cm below the water surface) in a circular pool for mice (Intex Recreation Corporation, Long Beach, CA; 91 cm in diameter, 40 cm in height, 25°C opaque water), located in a completely black-painted test room. Mice failing to find the platform were placed on it for 10 s, the same period as the successful animals. Animals were trained for four visible platform trials of 60 s per day and received the place navigation trial for 5 day. Each animal was submitted to sessions of four trials. The mouse was gently released (facing the wall) from one randomly selected starting point (east or west, as these were equidistant from the target) and allowed to swim until it escaped onto the hidden platform, which was now in a reversal position, located in the middle of the south quadrant. The time to find the platform was recorded as escape latency, and motion track and indexes were collected to evaluate the spatial learning ability of mice. The spatial probe trial was conducted on day 6. Duration of the NW quadrant-target quadrant within 60 s was observed, and the spatial memory ability of the mice was evaluated.

### 2.5. Western Blot Analysis

After the behavioral test, five mice randomly selected from each group were deeply anesthetized with 4% pentobarbital and then received a transcardiac perfusion of ice-cold sterilized saline (20 ml per mouse). The brain was taken out behind the ear, and then the hippocampus tissue was stripped and put into liquid nitrogen immediately, and finally preserved in the refrigerator at -80°C for use. After extracted and quantified, the supernatant of protein was denatured by boiling for 5 min in SDS sample buffer. 40 *μ*g of total protein was separated by 6%–15% SDS-PAGE, blotted onto PVDF membranes, and then probed with the following antibodies: monoclonal amyloid-*β* antibody (sc-28365, Santa Cruz Biotech), monoclonal anti-Phospho-p38 MAPK antibody (#9216, Cell Signaling Technology), rabbit anti-Tau (phospho, S262) antibody (ab64193, Abcam), rabbit anti-Synaptophysin antibody (ab14692, Abcam), rabbit anti-p38 antibody (ab170099, Abcam), mouse anti-Tau antibody (ab80579, Abcam), and mouse anti-GAPDH antibody (BM3876, Bosterbio), conjugated to horseradish peroxidase were used as secondary antibodies. Protein bands were visualized by incubation with BeyoECL Plus (P0018, Beyotime, China) for 1 min and imaged by a Gel Image System (Tanon, 5200, China). Densitometry was performed by using ImageJ software.

### 2.6. Immunofluorescence

The remaining five mice were randomly selected from among group for perfusion sampling and frozen brain sections. The mouse anti-amyloid antibody (sc-28365, Santa Cruz Biotech), mouse anti-Phospho-p38 MAPK antibody (#9216, Cell Signaling Technology), rabbit anti-Tau (phospho, S262) antibody (ab64193, Abcam), rabbit anti-Synaptophysin antibody (ab32594, Abcam), sheep anti-Iba1 antibody (ab5076, Abcam), and rabbits anti-Tmem antibody (ab209064, Abcam) were used in the immunofluorescence, performed following the previously described protocols [12]. Six image areas were randomly selected according to the hippocampal region, Image Proplus 6.0, which was used to analyze the cumulative optical density as well as the positive cell number in the selected visual area.

### 2.7. Morphological Assessment and Cell Counts

The Iba1 was used to identify microglia, and the morphologies of Iba1-positive cells were sorted into categories from ramified and amoeboid, as described in previous study [13]. Ramified cells had a diameter of 15+ *μ*m with fine and highly branched processes, while amoeboid cells had a diameter smaller than 10 *μ*m with retracted processes and irregular shapes. Cells were categorized in the hippocampal CA1. Iba1-positive cells were only counted if more than 75% of their staining was inside the boundaries of the counting frame and met the criteria of one of the different diameters. A Leica DM500 microscope with a high resolution digital camera (Leica MC120 HD) and LAS4.4 software was used for image acquisition using a 40x objective. All image groups were blinded to the researcher for cell counts and morphological quantification, and Iba1-positive cells within the entire 40x image frame were counted.

### 2.8. Statistical Analysis

The statistical analysis was performed using the SPSS 23.0, and the data were expressed as the mean ± SD. Since the data were normally distributedly tested by Kolmogorov-Smirnov (K-S), *T*-test is used to compare the two groups of mice in the open-field test. Furthermore, two-way ANOVA with repeated measures was used to analyze the different treatment in mice and data in different groups at the same time; in addition, data of spatial exploration experiment, WB, and IF were analyzed by one-way ANOVA.

## 3. Results

### 3.1. “Olfactory Three-Needle” Improved Spatial Learning and Memory Impairment of SAMP8 Mice

**Figure 1:**
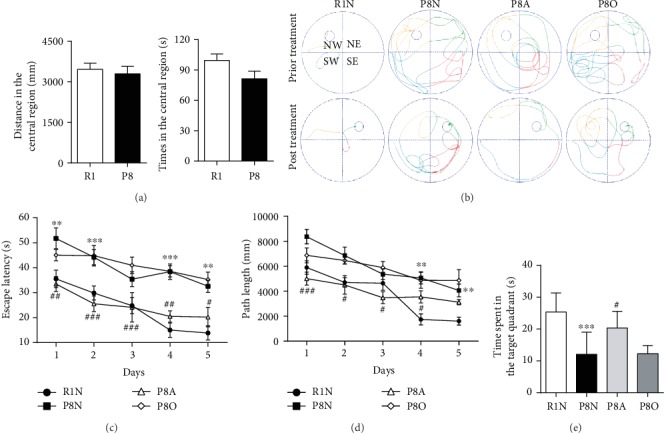
Effects of “olfactory three-needle” and olfactory stimulation on the behavior of SAMP8 mice. (a) Comparison of spontaneous activity between SAMR1 mice (R1) and SAMP8 mice (P8) in the open-field test. (b) Swimming trajectories in the SAMR1 normal (R1N) group, the SAMP8 normal (P8N) group, the SAMP8 acupuncture treatment (P8A) group, and the SAMP8 olfactory stimulation (P8O) group. (c) Comparison of the escape latency of all groups in the place navigation test. (d) Comparison of the path length to climb onto the platform of all groups in the place navigation test. (e) Comparison of the time spent in the target quadrant of all groups in the spatial probe test. ^∗∗^*P* < 0.01 and ^∗∗∗^*P* < 0.001 compared with the R1N group. ^#^*P* < 0.05, ^##^*P* < 0.01, and ^###^*P* < 0.001 compared with the P8N group.

Memory impairment commonly occurs in AD. To assess the potential neuroprotective effects of “olfactory three-needle” on memory impairment in SAMP8 mice, we subjected mice to the open-field test to measure the locomotor activity and exploratory behavior before “olfactory three-needle” manipulation, and then a Morris water maze to measure the ability of spatial learning and memory. In the open-field trial, there was no significant difference in distance and time in the central region between P8 and R1 mice (Figure 1(a)), indicating that the autonomous activity status of the two groups was similar before intervention. The swimming trajectories of R1 mice and P8 mice before and the first day after treatment are presented in Figure 1(b).

The results of the place navigation test in the Morris water maze test are presented in Figures 1(c) and 1(d); the P8N group showed significantly longer escape latency from day 1 to day 2 and day 4 to day 5 and longer path length to climb onto the platform on day 4 to day 5 than the R1N group, while the P8A group showed significantly shorter escape latency from day 1 to day 4 and the path length to climb onto the platform from day 1 to day 2 compared with the P8N group. In the spatial probe test (Figure 1(e)), the P8N group showed less time spent in the target quadrant than the R1N group, suggesting that the spatial memory ability of SAMP8 mice decreased. Furthermore, whether compared with the P8N group or the P8O group, the P8A group showed more time spent in the target quadrant in the spatial probe trial. However, there was no significant difference in behaviors between the P8O group and the P8N group. Those data suggested that “olfactory three-needle” could shorten the escape latency and path length of place navigation trial, prolonging the swim time in the platform quadrant in spatial probe trial to improve the spatial learning and memory ability of SAMP8 mice.

### 3.2. “Olfactory Three-Needle” and Olfactory Stimulation Rescued Amyloid *β* Protein Deposition and Hyperphosphorylated Tau Proteins in the Hippocampus of SAMP8 Mice

**Figure 2:**
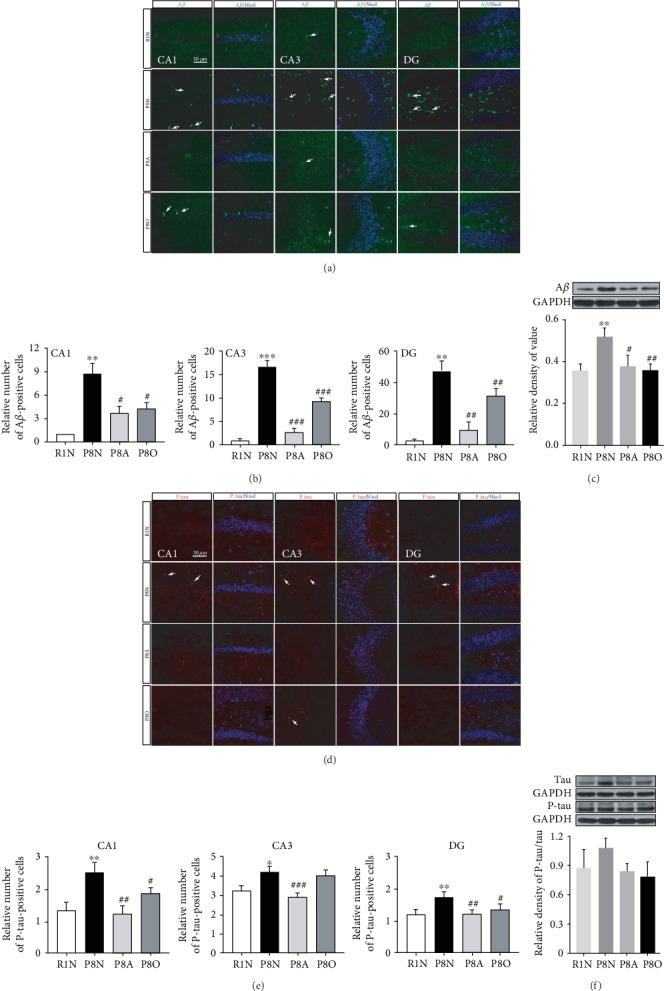
Effects of “olfactory three-needle” and olfactory stimulation on A*β* deposition and phosphorylation of tau protein in the hippocampus of SAMP8 mice. (a) Representative IF staining for A*β* in the hippocampus. A*β* in green and Nissl in blue; scale bar is 50 *μ*m. (b) Relative number of A*β*-positive cells in the CA1, CA3, and DG areas of the hippocampus. (c) Protein expression of A*β* expression in the hippocampus. (d) Representative IF staining for P-tau in the hippocampus. P-tau in red and Nissl in blue. (e) Relative number of P-tau-positive cells in the CA1, CA3, and DG areas of the hippocampus. (f) Protein expression and phosphorylation of tau protein in the hippocampus. ^∗^*P* < 0.05, ^∗∗^*P* < 0.01, and ^∗∗∗^*P* < 0.001 compared with the R1N group. ^#^*P* < 0.05, ^##^*P* < 0.01, and ^###^*P* < 0.001 compared with the P8N group.

To evaluate potential neuroprotective effects of acupuncture on learning and memory ability, the pathological A*β* deposition in the hippocampus of AD mice was measured. As expected, the relative number of A*β*-positive cells in the CA1, CA3, and DG areas of the hippocampus was dramatically increased in the P8N group compared with the R1N group, but significantly decreased by “olfactory three-needle” and olfactory stimulation (Figures 2(a) and 2(b)). The expression of A*β* protein in the hippocampus was significantly upregulated in the P8N group, while it was significantly downregulated in the P8A group and the P8O group (Figure 2(c)), indicating that “olfactory three-needle” and olfactory stimulation could inhibit the overdeposition of A*β* in the hippocampus of SAMP8 mice.

The deposition of A*β* protein induces intracellular accumulation of paired helical filaments consisting of hyperphosphorylated tau proteins [14]. The P-tau protein was found in the CA1, CA3, and DG areas of SAMP8 mouse hippocampus (Figure 2(d)). The relative numbers of P-tau-positive cell of the P8N group were significantly increased in the CA1, CA3, and DG regions, but the “olfactory three-needle” and olfactory stimulation decreased the number of hyperphosphorylated tau cells (Figure 2(e)). Interestingly, “olfactory three-needle” and olfactory stimulation both could reduce the protein expression ratio of P-tau to tau, but there was no significant difference among all the groups at the protein expression level of P-tau/tau in the hippocampus (Figure 2(f)). In general, “olfactory three-needle” and olfactory stimulation could inhibit abnormal phosphorylation of tau protein in different areas of the hippocampus.

### 3.3. The Effect of “Olfactory Three-Needle” on Inhibiting the Activation of p38 MAPK in the Hippocampus of SAMP8 Mice Was Irrelevant to the Stimulation of the Olfactory System

**Figure 3:**
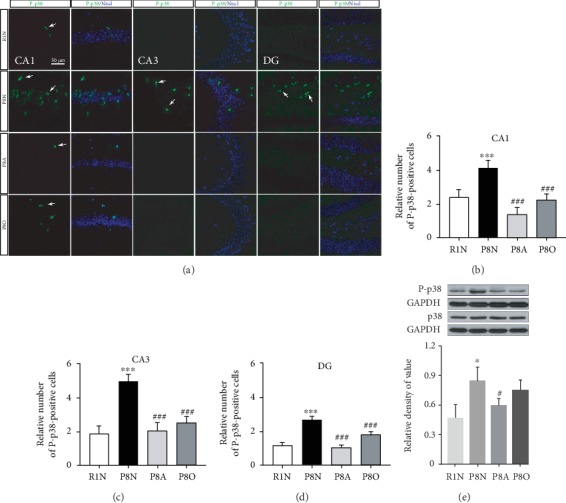
Effects of “olfactory three-needle” and olfactory stimulation on the activation of p38 MAPK in the hippocampus of SAMP8 mice. (a) Representative IF staining for P-p38 MAPK in the hippocampus. P-p38 in green, Nissl in blue. (b–d) Relative number of P-p38-positive cells in the CA1, CA3, and DG areas of the hippocampus. (e) Protein expression and activation level of p38 MAPK in the hippocampus. ^∗^*P* < 0.05, ^∗∗^*P* < 0.01, and ^∗∗∗^*P* < 0.001 compared with the R1N group. ^#^*P* < 0.05, ^##^*P* < 0.01, and ^###^*P* < 0.001 compared with the P8N group.

It is clear that A*β* deposition in AD brain induced neurotoxicity, resulting more serious nerve injuries. Furthermore, the activation of p38 MAPK is involved in mediating apoptosis; thus, the phosphorylated p38 MAPK was used to speculate on the effect of “olfactory three-needle” and olfactory stimulation on neuronal apoptosis. A large number of P-p38 MAPK-positive cells were observed in the hippocampal CA1, CA3, and DG of the P8N group (Figure 3(a)). The relative numbers of P-p38 MAPK-positive cells in the hippocampal CA1, CA3, and DG of the P8N group were significantly increased than those of the R1N group, while the relative numbers of P-p38 MAPK-positive cells in all regions of the hippocampus were both significantly decreased in the P8A group and the P8O group (Figures 3(b)–3(d)). The activation level of p38 MAPK protein (the ratio of P-p38 to p38) in the P8N group was significantly higher than that of the R1N group. However, only the P8A group significantly decreased the activation level of p38 MAPK compared with P8N, and there was no difference between the P8N group and the P8O group, suggesting that the effect of “olfactory three-needle” on inhibiting the activation of p38 MAPK was irrelevant to the stimulation of the olfactory system.

### 3.4. “Olfactory Three-Needle” and Olfactory Stimulation Contributed to the SYP Release of Hippocampal Neurons in SAMP8 Mice

Synaptic dysfunction was closely related to the impairment of spatial learning and memory, and the decrease of synaptophysin (SYP) release is the main reason for the synaptic dysfunction in AD patients [15]. Thus, the SYP released from the presynaptic membrane of hippocampal neurons was detected to corroborate the effect of olfactory three-needle and olfactory stimulation on synaptic function and neuronal survival.

**Figure 4:**
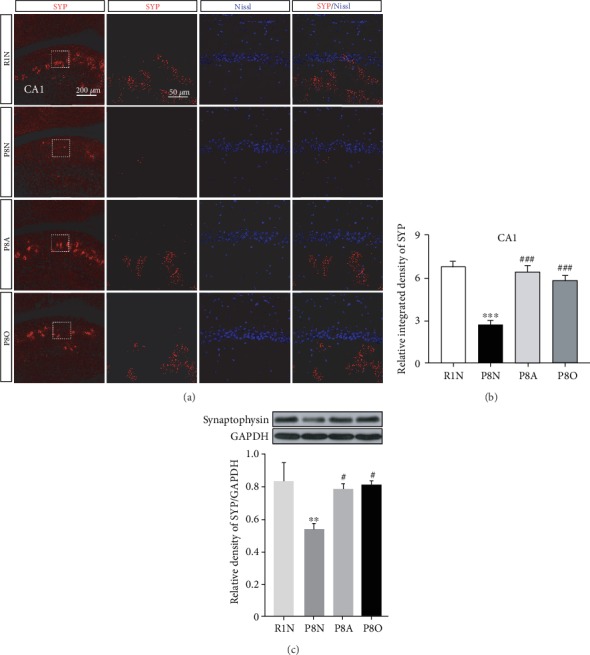
Effects of “olfactory three-needle” and olfactory stimulation on the recovery of synaptic function in hippocampal neurons of SAMP8 mice. (a) Representative IF staining for SYP in the hippocampus. SYP in red, Nissl in blue. (b) Relative integrated density of SYP in the CA1 area of the hippocampus. (c) Protein expression of SYP in the hippocampus. ^∗^*P* < 0.05, ^∗∗^*P* < 0.01, and ^∗∗∗^*P* < 0.001 compared with the R1N group. ^#^*P* < 0.05, ^##^*P* < 0.01, and ^###^*P* < 0.001 compared with the P8N group.

The SYP was observed to be widely distributed in the CA1 area of the hippocampus (Figure 4(a)). However, the relative integrated densities of SYP in the CA1 region and the SYP expression content in the hippocampus were both significantly reduced in the P8N group, compared with the R1N group (Figures 4(b) and 4(c)), while the relative integrated density of SYP in the CA1 region and the SYP expression content in the hippocampus were significantly improved by “olfactory three-needle” and olfactory stimulation (Figures 4(b) and 4(c)), suggesting that “olfactory three-needle” and olfactory stimulation played major roles in promoting SYP release and recovery of synaptic function in hippocampal neurons of SAMP8 mice.

### 3.5. “Olfactory Three-Needle” Played an Important Role in Antineuroinflammatory Reaction by Inhibiting the Activation of MG of SAMP8 Mice

The neuroinflammatory in the brain was mainly caused by activated microglia [16]. Whereas microglia in the brain were in a resting state, when stimulated by cerebral ischemia and nerve injury, microglia will be activated and accompanied by the morphological change from ramified to ameboid as a sign of microglial phagocytosis [17]. Iba1 can be invoked as a specific marker to display the activated microglia in brain tissue [12].

**Figure 5:**
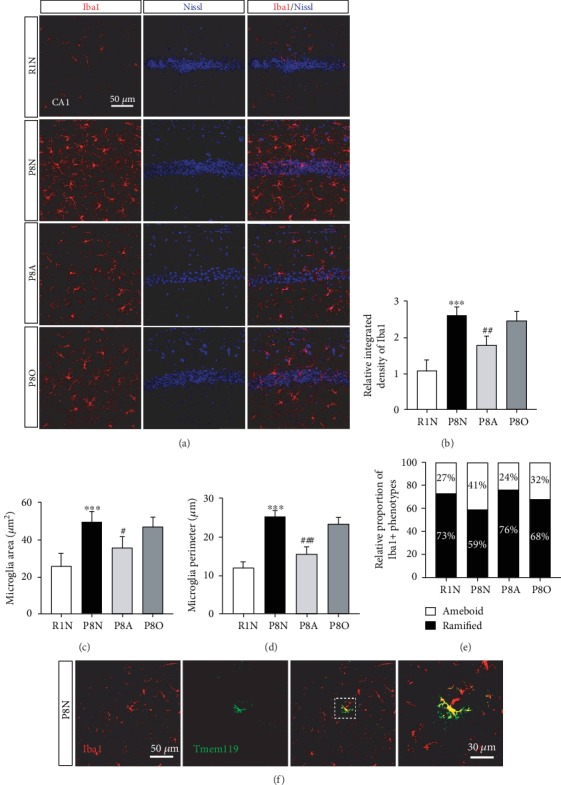
Effects of “olfactory three-needle” and olfactory stimulation on antineuroinflammatory reaction in hippocampal neurons of SAMP8 mice. (a) Representative IF staining for Iba1 to discriminate the ramified and ameboid MG in the hippocampus. Iba1 in red and Nissl in blue. (b) Relative integrated density of Iba1 in the CA1 area of the hippocampus. (c) The area of MG in the hippocampus. (d) The perimeter of MG in the hippocampus. (e) Relative proportion of activated (ameboid) and resting (ramified) MG in the hippocampus. (f) Coexpression of Iba1 and TMEM119. Iba1 in red, Tmem119 in green, and mergence in yellow. ^∗^*P* < 0.05, ^∗∗^*P* < 0.01, and ^∗∗∗^*P* < 0.001 compared with the R1N group. ^#^*P* < 0.05, ^##^*P* < 0.01, and ^###^*P* < 0.001 compared with the P8N group.

The representative IF staining for Iba1 showed that some of the microglia in the hippocampus were ramified (resting) and some were ameboid (activated) in the CA1 area of the hippocampus (Figure 5(a)). Comparing with the R1N group, the relative integrated density of Iba1+ cell was significantly higher in the P8N group, whereas the P8A group significantly reduced the relative integrated density of Iba1+ cell (Figure 5(b)). Furthermore, we investigated the activation of microglia by measuring the area and perimeter of microglia, and we found that the activated microglia in the P8N group had greater area and longer perimeter than that in the R1N group, which was significantly reduced by the treatment of “olfactory three-needle” (Figures 5(c) and 5(d)), reminding that “olfactory three-needle” played an important role in antineuroinflammatory reaction by inhibiting the activation of microglia of SAMP8 mice, and it was also confirmed by proportion of activated microglia in the hippocampus (Figure 5(e)).

Notably, TMEM119, as a distinctive marker of microglia, was expressed exclusively on a subset of Iba1+ CD68+ microglia with ramified and amoeboid morphologies in the brains of neurodegenerative diseases, such as Alzheimer's disease (AD) [18]. However, we found that there was few TMEM119 and Iba1 coexpression cells in the hippocampus of the SAMP8 mice, and the microglia coexpressed with TMEM119 and Iba1 was ramified (Figure 5(f)), which suggested that the subset of Iba1+ TMEM119+ microglia was not the main proinflammatory microglia in our study.

## 4. Discussion

Olfactory disorders are involved in neurodegenerative diseases, most probably during the early stages of AD, because of the early presence of olfactory identification problems [2]. As the main afferent for the limbic system, the entorhinal cortex receives direct sensory input from olfactory and relays information from the cortical association areas towards the hippocampal formation; therefore, olfactory probably regulates emotions and behaviors [19]. Although we have found that “olfactory three-needle” was able to improve memory impairment of AD patients in clinical [8], the therapeutic mechanism remains poorly understood.

SAMP8 mice presented age-related deterioration in behavior, and these mice exhibited aging rapidly as early as 4 months; accompanied by rapid-aging syndrome, learning and memory deteriorated at the age of 6 months and progressed with age [20, 21]. Furthermore, the abnormal expression protein and mRNA of *β*-amyloid precursor in the hippocampus increased with age [22], which were in line with the pathological features in the brain of AD patient. Moreover, since the neuropathological disorders of SAMP8 mice were similar to the naturally cerebral degenerative process, they can simulate the pathological manifestations of AD more comprehensively than other mice.

A*β*, as a theological polypeptide substance, accumulated pathologically in the brain of AD if without clearance timely [23]. Previous studies have demonstrated that the A*β*-induced neurotoxicity could result in a series of interrelated reactions, including hyperphosphorylated tau protein and microglia activation, which would lead to neuronal apoptosis and eventually spatial memory impairment [24, 25]. In our study, the SAMP8 mice showed obvious learning and memory impairment and excessive deposition of A*β* in the hippocampus, indicating that there was analogous pathological features in AD and SAMP8 mice [11]. “Olfactory three-needle” and olfactory stimulation with eugenol significantly reduced the A*β* deposition in the hippocampus. However, olfactory stimulation with eugenol did not improve the behavioral disorder of AD mice, but only the “olfactory three-needle” could shorten the escape latency and distance to climb onto the platform and prolong the duration in the target quadrant of SAMP8 mice in the Morris water maze test. Therefore, “olfactory three-needle” has great significance for reducing the A*β* deposition in the hippocampus and improving the spatial learning and memory disorder of the SAMP8 mice. Moreover, the data suggested that the mechanism of “olfactory three-needle” on SAMP8 mice was not the same as olfactory stimulus with eugenol.

The formation of neurofibrillary tangles (NFTs), as another pathological features in the brain of AD, is related to the abnormal phosphorylation of tau protein [26]. Aberrant phosphorylation of tau protein can cause changes in synaptic structure and function, synaptic loss, and neurotransmitter inactivation [27] and induce learning and memory impairment and loss of synaptic function in transgenic model mice [28]. A recent report has shown that the A*β* polypeptide fragments were involved in promoting the high phosphorylation of tau protein through mTOR signaling pathway to induce nerve apoptosis [29], while some studies have shown that the p38 MAPK signaling pathway may also contribute to the regulation of tau protein phosphorylation in the hippocampus [30, 31]. In our study, the neurons with high expression of P-p38 and P-tau were observed in the hippocampus CA1, CA3, and GD areas of SAMP8 mice. Interestingly, only the protein expression of P-p38/P38 increased significantly in the hippocampus of SAMP8 mice, but there was no significant difference in the protein expression of P-tau/tau between SAMP8 and SAMR1 mice. Although “olfactory three-needles” and eugenol could significantly reduce the number of P-tau and P-p38 positive neurons and the expression level of P-p38/p38 in the hippocampus of SAMP8 mice, the expression level of P-tau/tau was not changed by “olfactory three-needles” or olfactory stimulation with eugenol. Therefore, we inferred that the spatial learning disorder in SAMP8 mice used in our study was not caused by hyperphosphorylation of tau protein. However, whether “olfactory three-needles” or eugenol could regulate the phosphorylation of tau through the p38 MAPK pathway needs to be further studied.

The synaptic loss and apoptosis in neurons are also the important pathological features of AD patients [32]. It is reported that nearly half of the synaptophysin (SYP) in neocortex was lost in AD patients compared with healthy people [33]. The release of SYP played an important role in decreasing synaptic apoptosis and promoting synaptic plasticity [34]. The deposition of A*β* could interfere with synaptic regeneration and the release of SYP, resulting in the neurodegenerative lesions and spatial learning and memory disorder [35]. In our study, the expression of SYP in the hippocampus of SAMP8 mice was significantly decreased, which might be the main cause for synaptic impairment and memory loss. “Olfactory three-needles” and eugenol both could reduce A*β* deposition and promote SYP release, but the mere olfactory stimulus with eugenol did not improve the learning and memory disorder of the SAMP8 mice, indicating that the effect of “olfactory three-needles” on spatial learning and memory impairment of AD was not simply realized by stimulating olfactory nerve to promote SYP release and reduce A*β* deposition.

The neuroinflammation of AD is an immune response involving microglia (MG), and the activated MG has a dual regulatory effect on the protection of neurons [36]. Activated MG have the potential to clear A*β* by the phagocytosis [37] and release the neurotrophic factors to promote the formation of learning-dependent synapse [38]. Nevertheless, disproportionate activation of MG would secrete a large number of inflammatory factors, such as IL-1, IL-6, and TNF-*α*, to promote the A*β* deposition and induce neuronal apoptosis [39]. Moreover, the microglia-mediated A*β* neurotoxicity was closely related to the activation of MAPK, and attenuating p38MAPK phosphorylation made a contribution to protect A*β*-induced impairment of synaptic toxicity, memory dysfunction, and long-term potentiation in the entorhinal cortex [40]. Although both “olfactory three-needles” and eugenol could reduce the number of neurons expressing P-p38, only “olfactory three-needles” could significantly reduce p38MAKP phosphorylation and GM activation in the hippocampus of SAMP8 mice. Therefore, the “olfactory three-needle” could reduce the neuroinflammatory reaction by inhibiting p38MAPK phosphorylation and the excessive activation of MG in the hippocampus of the SAMP8 mice, which was of great importance for the removal of A*β* and the restoration of synaptic function.

In summary, the “olfactory three-needles” and mere olfactory stimulation with eugenol can reduce the A*β* deposition and promote the release of SYP, but only the “olfactory three-needles” can significantly improve the learning and memory disorder of SAMP8 mice, because the stimulation of olfactory nerves alone cannot improve the neuroinflammatory reaction. However, “olfactory three-needles” can improve the learning ability of SAMP8 mice by inhibiting p38MAPK phosphorylation and excessive activation of MG to decrease the neuroinflammatory response and A*β* neurotoxicity, which is closely related to the promotion of synaptic regeneration.

## Data Availability

The data used to support the findings of this study are available from the corresponding authors upon request.
